# The elderly's satisfaction with physical activity programs in senior welfare centers

**DOI:** 10.3389/fpubh.2023.1170612

**Published:** 2023-03-31

**Authors:** Yang-Hun Jung, Jeong-Beom Park, Austin Kang, Kuy-Chung Cho

**Affiliations:** ^1^Department of Sports Education, Hanshin University, Seoul, Republic of Korea; ^2^Department of Sports Education, Daelim University, Seoul, Republic of Korea; ^3^Department of Medicine, Seoul National University, Seoul, Republic of Korea

**Keywords:** elderly, user satisfaction, physical activity, program, senior welfare centers

## Abstract

**Introduction:**

Healthcare for the aging population has become a crucial issue in South Korea to maintain the elderly's quality of life, and physical activity is of primary importance for older adults. This study evaluated the exercise characteristics and satisfaction of the elderly who participated in physical activity programs provided by senior welfare centers in South Korea.

**Methods:**

We surveyed 266 participants to learn the characteristics of the elderly's exercise participation and their satisfaction with instructors, exercise programs, and facilities provided by senior welfare centers. A total of 263 copies were analyzed using the SPSS 23.0 statistical software.

**Results and discussion:**

The top three physical activity programs that the elderly participated in senior welfare centers were dancing (25.3%), gymnastics (24.8%), and table tennis/badminton (13.2%). There were significant differences in respondents' satisfaction according to sex, education level, spouse, family type, and monthly income per household (*p* < 0.05). The elderly were satisfied with programs (4.183 ± 0.483), facilities (3.881 ± 0.483), and instructors (3.604 ± 0.483) in order. Also, this study shows that user satisfaction differs depending on the demographic characteristics (gender, education, marital status, family type, economic status) and the characteristics of the exercise participation of the elderly (exercise duration, participation period).

**Conclusions:**

In conclusion, we presented the elderly's satisfaction with physical activity programs in senior welfare centers, suggesting that the elderly need physical activity programs according to their demographic and exercise characteristics.

## 1. Introduction

The proportion of older people in the population is reported to increase in all highly mechanized countries worldwide (1). Among the Organization for Economic Co-operation and Development (OECD) countries, South Korea is the fastest aging country (2). Healthcare for the aging population has become a crucial issue in South Korea to maintain older adults' quality of life, and physical activity is of primary importance for older adults (3). A strong connection has been noted between increased physical inactivity and chronic diseases (4). With age and physical inactivity, increased insulin resistance and decreased lipoprotein lipase activity in the skeletal musculature can lead to chronic diseases such as atherosclerosis (5), with follow-up effects such as myocardial insufficiency, coronary heart disease, hypertension, stroke, and type II diabetes (6, 7). Furthermore, higher physical activity levels are associated with better cognitive function in older adults (1). Being physically active and/or engaging in regular exercise are positively related to indicators of healthy aging (8). Since the aging process is associated with deterioration in several biological systems, the elderly are required to perform daily activities at a higher percentage of their maximum physiological reserve (9). Thus, regular physical activity can help improve physical and mental functions as well as reverse some effects of chronic disease to keep older people mobile and independent (10). In addition, successful aging is an important concept for describing the quality of aging (11). Successful aging can be defined as high physical, psychological, and social functioning in old age without major diseases (12). Moreover, physical activity is known to support successful aging (13). The more active people are in their later years, the greater their life satisfaction (14). Furthermore, physical activity is associated with improved quality of life among older individuals (15).

Although regular physical activity is essential for maintaining long-term physical, cognitive, and emotional health for the elderly, few older adults engage in routine physical activity (16). For example, ~80% of Korean older adults aged 60–70 years do not engage in moderate physical activity and 90% of older adults over 71 years of age are inactive (17). Also, it is clear that the elderly become cognizant of their age-related physical limitations, and their awareness radiates into a lack of confidence in their abilities (18). They are likely to have uncertainty about what activities are safe, as well as fear of injury, pain, overexertion, or prolonged recovery. For this reason, lack of professional guidance (e.g., instructors, programs, and facilities) served as a major barrier to physical activity in general (16). In particular, health programs provided by senior welfare centers and community facilities contribute to increased physical activity and promote improved quality of life among the elderly (19). Meanwhile, participation in physical activity in the elderly can be influenced by a number of variables including demographic factors such as gender, education, and marital status (17). For example, physical activity participation is lower among older females (20) and less educated older seniors (21). Choices of older adults to be regularly physically active are also influenced by social support from family members or friends, availability of facilities for exercise and/or recreational activities, personal determinants especially one's motivation, self-efficacy, and self-regulation skills (17). In particular, user participation is related to user satisfaction (18). User satisfaction is a term frequently used in marketing (22). Satisfaction is defined as an effective statement about emotional reactions to the experience of products and services, which is influenced by user satisfaction with these products or services and by the information used to select products or services (23). Therefore, in order to increase the participation rate of programs promoting physical activity among the elderly attending senior welfare centers, an evaluation of user satisfaction will be needed. Studies on physical activity with seniors have mostly focused on programs, mental health, including depression and anxiety, and demographic factors (17, 24, 25). However, few studies have analyzed the reasons for not participating in physical activities provided by senior centers in terms of user satisfaction. Considering this, we analyzed the elderly's satisfaction with physical activity programs in senior welfare centers in Korea. Thus, this study was conducted to contribute to the promotion of physical activity of the elderly by increasing their satisfaction with the program of senior welfare centers.

## 2. Materials and methods

### 2.1. Research design

This descriptive study examined the user satisfaction of the elderly aged 65 years or older who participated in exercise programs of senior welfare centers in South Korea during the COVID-19 pandemic. The null hypothesis (*H*_0_) is that the demographic characteristics and the characteristics of the exercise participation of the elderly are unrelated to user satisfaction. Therefore, this study presents and verifies the following alternative hypotheses:

***H_1_***. *User satisfaction will differ depending on the demographic characteristics of the elderly*.
***H_2_***. *User satisfaction will differ depending on the characteristics of the exercise participation of the elderly*.

### 2.2. Participants

This study was conducted in compliance with the ethical issues presented in the Declaration of Helsinki, according to the guidelines of the Korean government that general surveys are not subject to review by the institutional review board. Thus, this study was conducted after obtaining voluntary consent from the elderly at two senior welfare centers in Seoul or Suwon, South Korea. The minimum sample size was obtained using G^*^power 3.1.9.2, based on previous studies (26, 27). With a significance level of 0.05, power of 0.95, and an effect size of 0.30, the minimum sample size was confirmed to be 242. Therefore, 266 participants were recruited, considering a dropout probability of 10 percent. Among these, three responses deemed insincere were excluded, resulting in 263 valid responses for analysis.

Table 1 shows participants' demographic characteristics. Of the 263 participants in this study, 154 (58.6%) were women, and 82 (31.2%) were between 71 and 75 years of age. Moreover, 151 (57.4%) had a spouse, and 54 (20.5%) respondents were living alone. One hundred thirty-three people (50.6%) had a monthly income per household of <1 million won (about $700). This suggests that some elderly adults are lonely or economically poor.

**Table 1:** Participants' demographic characteristics.

**Variable**	**Category**	**Frequency (*n*)**	**Percentage (%)**
Gender	Male	109	41.4
	Female	154	58.6
Age (years)	66–70	55	20.9
	71–75	82	31.2
	76–80	73	27.7
	≥81	53	20.2
Education level	Middle school graduate or lower	130	49.4
	High school graduate	95	36.1
	University graduate or higher	38	14.5
Presence of a spouse	Yes	151	57.4
	No	112	42.6
Family type	Living alone	54	20.5
	Living with direct descendants (one generation)	112	42.5
	Living with direct descendants (two generations)	70	26.7
	Living with direct descendants (three generations)	28	10.3
Monthly income per household	1,000,000 KRW or less	133	50.6
	1,000,001–2,000,000 KRW	47	17.9
	2,000,001–3,000,000 KRW	43	16.3
	3,000,001–4,000,000 KRW	20	7.6
	4,000,001 KRW or more	20	7.6
Total		263	100

KRW is the Korean currency unit. One dollar (USD) is equivalent to about 1,300KRW (as of August 2022).

### 2.3. Research instrument

A questionnaire was developed based on previous studies (28–30). The questionnaire contained 29 items, which consisted of three sections of questions related to the following dimensions: demographic variables, exercise participation characteristics, and user satisfaction. Responses were scored on a Likert scale ranging from 1 (“strongly disagree”) to 5 (“strongly agree”). An exploratory factor analysis was performed to analyze the validity of the instrument, and Cronbach's α was extracted for the analysis (Table 2). The principal component method was used for exploratory factor analysis to estimate factor loading, and the varimax method was selected as the rotation method.

**Table 2:** Results of the validity and reliability analysis.

**Category**	**Item**	**Rotated component matrix (varimax)**		
		**Factor 1**	**Factor 2**	**Factor 3**
Satisfaction with instructors	Instructors' counseling experience	0.749	0.151	0.063
	Appropriate assessment of instructors	0.893	0.156	0.134
	Trust in instructors	0.844	0.124	0.111
	Instructors' exact knowledge	0.845	0.031	0.206
	Kind manner of instructors	0.724	0.052	0.217
Satisfaction with programs	Segmentation of programs	0.051	0.914	0.192
	Suitability of programs	0.023	0.776	0.215
	Benefits of programs	0.159	0.851	0.167
	Diversity of programs	0.173	0.762	0.218
	Appropriate program fees	0.116	0.921	0.111
Satisfaction with facilities	Equipment of facilities	0.167	0.138	0.798
	Cleanliness in facilities	0.154	0.048	0.803
	Sufficient space in facilities	0.201	0.127	0.812
	Transportation to facilities	0.167	0.103	0.734
	Changing rooms and shower rooms in facilities	0.104	0.252	0.791
	Information on use of facilities	0.148	0.113	0.763
Eigenvalues		7.932	1.638	1.301
Variance %		49.671	9.892	7.668
Accumulated %		49.671	59.563	67.231
Cronbach's α		0.904	0.872	0.856

Kaiser-Meyer-Olkin test = 0.852.

Bartlett's test = 4.738 (p < 0.001).

The results demonstrated that all factors had a loading value of 0.7 or more for each questionnaire item, and the eigenvalue of each factor exceeded 1.0. In addition, Cronbach's αs for all items exceeded 0.8, indicating high reliability. Furthermore, Kaiser-Meyer-Olkin (KMO) is a value indicating the degree to which the correlation between variables is well explained by other variables. Because the KMO values of each variable are all >0.7, it can be interpreted that the selection of variables was good. Furthermore, the results of Bartlett's test indicated that the factor analysis model was suitable.

### 2.4. Data collection and analysis

The survey was conducted from August 1 to August 31, 2022, using a convenience sampling of South Korean men and women aged 65 years or older who regularly visited senior welfare centers during the COVID-19 pandemic. Written informed consent was obtained from the respondents, and data were collected using a structured questionnaire. A total of 263 copies were used for data analysis and data analysis was conducted through Statistical Package for the Social Sciences (SPSS) 23.0 statistical software (IBM, Armonk, NY, USA). Descriptive statistics such as mean, standard deviation, and frequency distribution were used at the descriptive level. In addition, one-way analysis of variance (ANOVAs) and Scheffé's *post hoc* pairwise comparison analyses were performed. Significance was set at 0.05.

## 3. Results

### 3.1. Participants' exercise participation characteristics

Table 3 shows the exercise participation characteristics of the respondents. Ninety-three respondents (35.3%) answered that they exercised 4–5 times a week at senior welfare centers. One-hundred twenty-eight respondents (48.6%) said they practiced 31 to 60 min per session. Ninety-one respondents (34.6%) answered that the exercise duration was “13–36 months.” A total of 204 (77.5%) respondents replied that healthcare was the primary purpose for participating in the physical activity program. Lastly, 168 (63.8%) answered that they mainly obtained information through word of mouth.

**Table 3:** Respondents' exercise participation characteristics.

**Variable**	**Category**	**Frequency (*n*)**	**Percentage (%)**
Exercise frequency	Once a week	9	3.4
	2–3 times a week	81	30.7
	4–5 times a week	93	35.3
	6–7 times a week	80	30.4
Exercise duration	30 min or less	35	13.3
	31–60 min	128	48.6
	61–90 min	38	14.4
	91–120 min	30	11.4
	121 min or more	32	12.1
Participation period	12 months or less	70	26.6
	13–36 months	91	34.6
	37–60 months	52	19.7
	61 months or more	50	19.1
Exercise purpose	Healthcare	204	77.5
	Physical rehabilitation	11	4.1
	Use of leisure time	30	11.4
	Other (friending, relieving stress, etc.)	18	6.8
Method of obtaining	Leaflet advertising	32	12.1
	information	Mobile advertising	27	10.2
	Word of mouth	168	63.8
	Other	36	13.6
Total		263	100

In addition, the results of the multiple response frequency analysis to determine what programs the elderly who participate in physical activity programs at the senior welfare center most frequently participate in. The top three physical activity programs were dancing (25.3%), gymnastics (24.8%), and table tennis/badminton (13.2%).

### 3.2. Analysis of satisfaction differences by demographic characteristics

Table 4 presents participants' satisfaction according to their demographic variables. In order, participants were satisfied with programs, facilities, and instructors, showing the results of the one-way ANOVA for satisfaction by demographic characteristics. There were significant differences in respondents' satisfaction according to sex, education level, spouse, family type, and monthly income per household. First, concerning gender, satisfaction with instructors was higher among men (3.672 ± 0.464) than women (3.527 ± 0.717; *p* < 0.001), satisfaction with programs was higher among women (4.314 ± 0.579) than men (4.042 ± 0.474; *p* = 0.001), and satisfaction with facilities was higher among women (3.971 ± 0.482) than men (3.793 ± 0.382; *p* = 0.038). Second, concerning age, there were no significant differences in satisfaction with instructors, programs, or facilities. Third, concerning education level, there was no difference in satisfaction with instructors; however, satisfaction with the programs (4.304 ± 0.594 vs. 3.758 ± 0.458; *p* < 0.001) and satisfaction with facilities (4.037 ± 0.338 vs. 3.657 ± 0.426; *p* = 0.002) were significantly higher in the low education group than in the high education group, respectively. These results were confirmed using Scheffé's *post-hoc* test. Fourth, concerning the presence of a spouse, satisfaction with the instructor was significantly higher in the group without a spouse (3.872 ± 0.713) than in the group with a spouse (3.463 ± 0.589; *p* = 0.001), satisfaction with the program was significantly higher in the group without a spouse (4.313 ± 0.634) than in the group with a spouse (4.123 ± 0.672; *p* = 0.018), and satisfaction with facilities was significantly higher in the group without a spouse (4.051 ± 0.627) than in the group with a spouse (3.789 ± 0.463; *p* = 0.003). Fifth, concerning family type, those living for three generations had higher satisfaction with the instructors (4.046 ± 0.589; *p* < 0.001), programs (4.388 ± 0.672; *p* = 0.007), and facilities (4.112 ± 0.463; *p* = 0.001) than did their counterparts. These results were confirmed by Scheffé's *post-hoc* test. Sixth, concerning monthly income per household, those who made 3,000,001–4,000,000 won had higher satisfaction with instructors (4.219 ± 0.723; *p* = 0.002) as compared to the other groups. Those who made 4,000,000 won or more were most satisfied with the program (4.468 ± 0.483; *p* = 0.046) as compared to the other groups. However, there was no significant difference in satisfaction with facilities among the groups.

**Table 4:** Results of the one-way analysis of variance (ANOVA) for user satisfaction with the demographic characteristics of the elderly.

**Category**		**Subcategory**	**Mean ±standard deviation**	***F***	***P***	***Post-hoc***
Gender	Satisfaction with instructors	Male^a^	3.672 ± 0.464	22.271	<0.001^***^	a>b
		Female^b^	3.527 ± 0.717						
	Satisfaction with programs	Male^a^	4.042 ± 0.474	10.923	0.002^**^	b>a
		Female^b^	4.314 ± 0.579			
	Satisfaction with facilities	Male^a^	3.793 ± 0.382	4.903	0.038^*^	b>a
		Female^b^	3.971 ± 0.482			
Age	Satisfaction with instructors	66–70^a^	3.781 ± 0.445	1.453	0.229	(-)
		71–75^b^	3.464 ± 0.436			
		76–80^c^	3.621 ± 0.522			
		≥81^d^	3.763 ± 0.533			
	Satisfaction with programs	66–70^a^	4.127 ± 0.724	0.493	0.726	(-)
		71–75^b^	4.201 ± 0.733			
		76–80^c^	4.208 ± 0.488			
		≥81^d^	4.268 ± 0.778			
	Satisfaction with facilities	66–70^a^	3.801 ± 0.718	1.509	0.223	(-)
		71–75^b^	3.824 ± 0.661			
		76–80^c^	3.946 ± 0.583			
		≥81^d^	4.029 ± 0.421			
Education level	Satisfaction with instructors	Middle school graduate or lower^a^	3.654 ± 0.532	2.754	0.077	(-)
		High school graduate^b^	3.743 ± 0.459			
		University graduate or higher^c^	3.346 ± 0.495			
	Satisfaction with programs	Middle school graduate or lower^a^	4.304 ± 0.594	12.387	<0.001^***^	a > b > c
		High school graduate^b^	4.246 ± 0.784			
		University graduate or higher^c^	3.758 ± 0.458			
	Satisfaction with facilities	Middle school graduate or lower^a^	4.037 ± 0.338	6.701	0.002^**^	a > b > c
		High school graduate^b^	3.792 ± 0.632			
		University graduate or higher^c^	3.657 ± 0.426			
Presence of a spouse	Satisfaction with instructors	Yes^a^	3.463 ± 0.589	11.791	0.002^**^	b>a
		No^b^	3.872 ± 0.713			
	Satisfaction with programs	Yes^a^	4.123 ± 0.672	5.587	0.018	(-)
		No^b^	4.313 ± 0.634			
	Satisfaction with facilities	Yes^a^	3.789 ± 0.463	9.142	0.003^**^	b>a
		No^b^	4.051 ± 0.627			
Family type	Satisfaction with instructors	Living alone^a^	3.891 ± 0.723	7.728	<0.001^***^	d >a >c > b
		Living with direct descendants (one generation)^b^	3.322 ± 0.483			
		Living with direct descendants (two generations)^c^	3.754 ± 0.495			
		Living with direct descendants (three generations)^d^	4.046 ± 0.589			
	Satisfaction with programs	Living alone^a^	4.377 ± 0.723	3.898	0.007^**^	d > a > c > b
		Living with direct descendants (one generation)^b^	4.068 ± 0.483			
		Living with direct descendants (two generations)^c^	4.179 ± 0.458			
		Living with direct descendants (three generations)^d^	4.388 ± 0.672			
	Satisfaction with facilities	Living alone^a^	4.109 ± 0.481	4.832	0.003^**^	d >a >c > b
		Living with direct descendants (one generation)^b^	3.739 ± 0.416			
		Living with direct descendants (two generations)^c^	3.887 ± 0.426			
		Living with direct descendants (three generations)^d^	4.112 ± 0.463			
Monthly income per household	Satisfaction with instructors	1,000,000 KRW or less^a^	3.467 ± 0.495	4.931	0.002^**^	d >e >c >b > a
		1,000,001–2,000,000 KRW^b^	3.501 ± 0.589			
		2,000,001–3,000,000 KRW^c^	3.849 ± 0.713			
		3,000,001–4,000,000 KRW^d^	4.219 ± 0.723			
		4,000,001 KRW or more^e^	4.018 ± 0.483			
	Satisfaction with programs	1,000,000 KRW or less^a^	4.157 ± 0.458	2.436	0.046^*^	e> d >c >a >b
		1,000,001–2,000,000 KRW^b^	4.068 ± 0.672			
		2,000,001–3,000,000 KRW^c^	4.209 ± 0.634			
		3,000,001–4,000,000 KRW^d^	4.221 ± 0.723			
		4,000,001 KRW or more^e^	4.468 ± 0.483			
	Satisfaction with facilities	1,000,000 KRW or less^a^	3.892 ± 0.426	2.217	0.067	(-)
		1,000,001–2,000,000 KRW^b^	3.749 ± 0.463			
		2,000,001–3,000,000 KRW^c^	3.838 ± 0.627			
		3,000,001–4,000,000 KRW^d^	4.168 ± 0.481			
		4,000,001 KRW or more^e^	4.159 ± 0.416			

^*^p < 0.05, ^**^p < 0.01, ^***^p < 0.001.

### 3.3. Analysis of satisfaction differences by exercise participation characteristics

Table 5 presents the means and standard deviations for each satisfaction level, based on respondents' exercise participation characteristics, showing the results of the one-way ANOVA for satisfaction by exercise participation characteristics. Overall, participants were satisfied with programs (4.183 ± 0.483), facilities (3.881 ± 0.483), and instructors (3.604 ± 0.483) in order. In this study, the following results were derived. First, concerning exercise frequency, there were no significant differences between groups for satisfaction with instructors, programs, or facilities. Second, concerning exercise duration, those in the “91–120 min” group had more satisfaction with instructors (3.873 ± 0.459) than those in the other groups, while those in the “ ≤ 30 min” group (2.943 ± 0.522) had the lowest level of satisfaction with instructors. This was verified through Scheffé's *post-hoc* test. There were no significant differences between groups for satisfaction with programs or facilities. Third, concerning the participation period, those in the “37–60 months” group had greater satisfaction with instructors (3.922 ± 0.723) as compared to those in the other groups, while the “ ≤ 12 months” group (2.943 ± 0.522) had the lowest level of satisfaction with instructors. This was verified through Scheffé's *post-hoc* test. Moreover, those in the “13–36 months” group had greater satisfaction with programs (4.312 ± 0.634) as compared to those in the other groups, while the “ ≤ 12 months” group (4.034 ± 0.672) scored the lowest. This was verified through Scheffé's *post-hoc* test. There was no significant difference in satisfaction with facilities among the groups.

**Table 5:** Results of the one-way analysis of variance (ANOVA) for user satisfaction with the characteristics of the exercise participation of the elderly.

**Category**		**Subcategory**	**Mean ±standard deviation**	***F***	***P***	***post-hoc***
Exercise frequency	Satisfaction with instructors	Once a week^a^	3.672 ± 0.464	1.512	0.211	(-)
		2–3 times a week^b^	3.527 ± 0.717			
		4–5 times a week^c^	3.813 ± 0.445			
		6–7 times a week^d^	3.544 ± 0.436			
	Satisfaction with programs	Once a week^a^	4.042 ± 0.474	1.523	0.209	(-)
		2–3 times a week^b^	4.073 ± 0.579			
		4–5 times a week^c^	4.276 ± 0.724			
		6–7 times a week^d^	4.271 ± 0.733			
	Satisfaction with facilities	Once a week^a^	3.743 ± 0.371	2.284	0.098	(-)
		2–3 times a week^b^	3.712 ± 0.482			
		4–5 times a week^c^	3.981 ± 0.718			
		6–7 times a week^d^	3.834 ± 0.661			
Exercise duration	Satisfaction with instructors	≤ 30 min^a^	2.943 ± 0.522	5.913	<0.001***	d> c>b>e> a
		31–60 min^b^	3.747 ± 0.533			
		61–90 min^c^	3.817 ± 0.532			
		91–120 min^d^	3.873 ± 0.459			
		≥121 min^e^	3.432 ± 0.495			
	Satisfaction with programs	≤ 30 min^a^	3.961 ± 0.488	1.983	0.098	(-)
		31–60 min^b^	4.214 ± 0.778			
		61–90 min^c^	4.146 ± 0.594			
		91–120 min^d^	4.377 ± 0.784			
		≥121 min^e^	4.342 ± 0.458			
	Satisfaction with facilities	≤ 30 min^a^	3.712 ± 0.583	1.139	0.344	(-)
		31–60 min^b^	3.922 ± 0.421			
		61–90 min^c^	3.931 ± 0.338			
		91–120 min^d^	4.011 ± 0.632			
		≥121 min^e^	3.928 ± 0.426			
Participation period	Satisfaction with instructors	≤ 12 months^a^	3.313 ± 0.589	4.753	0.004**	c>b >d>a
		13–36 months^b^	3.752 ± 0.713			
		37–60 months^c^	3.922 ± 0.723			
		≥61 months^d^	3.512 ± 0.483			
	Satisfaction with programs	≤ 12 months^a^	4.034 ± 0.672	2.587	0.049*	b>c>d> a
		13–36 months^b^	4.312 ± 0.634			
		37–60 months^c^	4.281 ± 0.723			
		≥61 months^d^	4.192 ± 0.483			
	Satisfaction with facilities	≤ 12 months^a^	3.843 ± 0.463	1.031	0.382	(-)
		13–36 months^b^	3.901 ± 0.627			
		37–60 months^c^	4.094 ± 0.481			
		≥ 61 months^d^	3.828 ± 0.416			

^*^p < 0.05, ^**^p < 0.01, ^***^p < 0.001.

### 3.4. Discussion

Physical activity can be defined as voluntary body movements which are produced by skeletal muscles, resulting in the increase in energy consumption (31). Physical activity has been a key part of active aging and the association between exercise and physical health is well established (19). In particular, exercise at advanced ages is important for maintaining physical fitness, promoting mobility, preventing falls, and providing access to opportunities that help personal independence (32). It is also reported that regular exercise had significant effects on elderly's self-consistency (33), and physical activity was significantly related to life satisfaction and happiness in the elderly (34, 35). Moreover, physical activity can improve quality of life and wellbeing of the elderly when compared with minimal or no-treatment controls (36). Previous studies suggested that the elderly in urban areas used the public exercise facilities regardless of their perceived health and that they preferred low-intensity exercises (37, 38). While reviewing these literature on the necessity of physical activity for the elderly, we paid attention to senior welfare centers in Korea. Senior welfare centers, also commonly known as senior centers, elderly centers or seniors' clubs, offer a wide variety of programs and services (39). By offering opportunities for social interaction and friendship, senior welfare centers have traditionally had a central role in easing loneliness, increasing social integration and reducing isolation (19, 40). In addition, institutions similar to the senior welfare centers in Korea have been operating in many developed countries to promote the welfare of the elderly. For example, ~15,000 community senior centers in the United States have given a wide range of services for seniors to improve their overall health and wellness in their community (41, 42). In this regard, there is a need to investigate users' satisfaction with the exercise programs provided to promote the health of the elderly in senior welfare centers.

This study examined the satisfaction of South Korean senior citizens (aged 65 years or older, mean age: 74.7 ± 1.483) with the physical activity programs provided by senior welfare centers during the COVID-19 pandemic. We evaluated user satisfaction with instructors, programs, and facilities according to demographic factors such as gender, age, education, presence of a spouse, family type, and monthly income per household attending senior welfare centers. This study confirmed that user satisfaction could be different according to all demographic factors except the age of the elderly. This finding indirectly supports the results of previous studies (17, 20, 21), which showed that a number of variables, including demographic factors such as gender, education, and marital status, could influence the participation in physical activity of the elderly. The most common responses for exercise participation at the senior welfare centers were exercising 4–5 times a week (35.3%), for 31–60 min (48.6%), and for over 13–36 months (34.6%). The top three physical activity programs that the elderly participated in senior welfare centers were dancing (25.3%), gymnastics (24.8%), and table tennis/badminton (13.2%). These preferences may vary depending on the size of the facility of the senior welfare center (e.g., swimming), but the top three exercise programs could be preferred by the respondents as they are suitable for the elderly with low strength and agility. In particular, dancing is an effective physical activity for improving static and dynamic balance control in the elderly, which is consistent with the previous study (43).

This study shows that user satisfaction differs depending on the demographic characteristics (gender, education, marital status, family type, economic status). Particularly, females were significantly less satisfied with instructors (3.527 ± 0.717) than males(3.672 ± 0.464), which was statistically significant (*p* < 0.001). Of the 263 participants in this study, 154 (58.6%) were female, and 109 (41.4%) were male. This finding is inconsistent with the results of Marquet et al. (20), who reported that physical activity participation was lower among older females. It could be related to the selection bias since we included only subjects attending two senior welfare centers located in the metropolitan area in Korea. The elderly with a higher level of education showed significantly lower satisfaction with exercise programs (3.758 ± 0.458) and facilities (3.657 ± 0.426) than other groups. In addition, it was found that the elderly with spouses were less satisfied with instructors (3.463 ± 0.589) and facilities (3.789 ± 0.463). The elderly living with direct descendants (one generation) showed the lowest level of satisfaction with instructors (3.322 ± 0.483), exercise programs (4.068 ± 0.483), and facilities (3.739 ± 0.416) during physical activities at senior welfare centers (*p* < 0.01). We also confirmed that the elderly with low household incomes (2 million won or less) were less satisfied with instructors (3.467 ± 0.495) or programs (4.068 ± 0.672). Our findings support the results of Tsou and Liu (44) who presented that individuals with a low income or who are unemployed have lower life satisfaction. This study also shows that user satisfaction can differ depending on the characteristics of the exercise participation of the elderly. For example, user satisfaction with the instructors was significantly lower in the elderly group (2.943 ± 0.522) who exercised <30 min (*p* < 0.001). These findings suggest that instructors' roles should be changed for the elderly with 30 min of exercise. In addition, it was confirmed that satisfaction with the instructors (3.313 ± 0.589) and exercise programs (3.313 ± 0.589) was relatively lower in the exercise group for <1 year compared to other groups. It was also confirmed that the elderly who attended senior welfare centers for more than 5 years were relatively less satisfied with the instructors (3.512 ± 0.483) and exercise programs (4.192 ± 0.483) than other groups. Therefore, it can be seen that the selection of instructors and changes in teaching methods or exercise programs are required for the elderly who have participated for more than 5 years. In our study, exercise frequency and exercise intensity were not related to user satisfaction of the elderly with exercise programs or facilities. This did not match the research results of An et al. (34), which reported that participants with a higher physical activity level tended to have higher life satisfaction and happiness, which could be related to their different indicators (life satisfaction vs. user satisfaction).

Based on the results, this study could suggest the following improvement plans. First, more diverse methods should be developed for the elderly to obtain information on senior welfare center programs. Second, it is necessary to find a way to increase older men's satisfaction with physical activity programs at senior welfare centers, since they prefer more active exercise than women. Third, senior welfare centers had better develop segmented programs according to the elderly's physical strength and age, providing appropriate exercise instructors and secure safe and convenient facilities for the elderly. However, since muscle weakness in the elderly threatens health (45), it is necessary to consider exercises to strengthen the muscles of the elderly when organizing exercise programs in senior welfare centers. Second, there were differences in user satisfaction according to sex, education level, spouse, family type, and household income. However, the difference in satisfaction between the groups according to age was not significant. It has been reported that life satisfaction has a negative propensity with age, which differs from the results of this study—that exercise satisfaction is independent of age (46). Third, the differences in satisfaction among the groups according to exercise duration and participation period were significant. However, there was no significant difference among the groups in satisfaction with exercise frequency. Therefore, careful consideration is required for elderly individuals who regularly exercise at senior welfare centers. Based on these results, the hypotheses of this study could be verified as follows. First, hypothesis 1, that user satisfaction will differ depending on the demographic characteristics of the elderly, is partially accepted. Second, hypothesis 2, that user satisfaction will differ depending on the characteristics of the exercise participation of the elderly, is also partially accepted.

## 4. Conclusions

This study was conducted to identify problems and improvement plans for physical activity programs in senior welfare centers considering participants' satisfaction with the instructors, programs, and facilities. We recruited 266 participants attending two senior welfare centers in a metropolitan area in Korea, and 263 valid responses were analyzed. As a result, we report that user satisfaction differs depending on the demographic characteristics including gender, education, marital status, family type, and economic status, presenting the characteristics of the exercise participation of the elderly, such as exercise duration and participation period. This study also shows a strategy for physical activity programs in senior welfare centers, suggesting that it is necessary to provide physical activity programs for the elderly in senior welfare centers according to their demographic and exercise characteristics. Thus, this research is differentiated from other studies in that it evaluated the elderly's user satisfaction with instructors, exercise programs, and facilities according to demographic characteristics and the characteristic of the exercise participation, suggesting the need to improve items with low user satisfaction among the elderly.

This study contributed to the literature by identifying and examining user satisfaction that plays a role in facilitating or hampering the elderly's participation in senior welfare centers. However, there is a limitation in generalization in that it investigated only physical activity programs provided at two senior welfare centers in the Seoul metropolitan area during the COVID-19 pandemic. Thus, follow-up studies should be conducted, including various ages and regions. Nevertheless, this study is meaningful in that it investigated the exercise characteristics and satisfaction of the elderly attending physical activity programs at senior welfare centers and suggested improvement plans.

## Data availability statement

The data used to support the findings of this study are included within the article.

## Author contributions

Y-HJ has made substantial contributions to conception, design, acquisition of data, and writing the original draft. J-BP contributed to analysis and interpretation of data. AK and K-CC contributed to supervision, review, and editing. All authors contributed to the article and approved the submitted version.
